# Genistein treatment improves fracture resistance in obese diabetic mice

**DOI:** 10.1186/s12902-016-0144-4

**Published:** 2017-01-09

**Authors:** Britton Odle, Nathan Dennison, Layla Al-Nakkash, Tom L. Broderick, Jeffrey H. Plochocki

**Affiliations:** 1 Arizona College of Osteopathic Medicine, Midwestern University, Glendale, AZ USA; 2 Department of Physiology, Arizona College of Osteopathic Medicine, Midwestern University, Glendale, AZ USA; 3 Department of Physiology, Laboratory of Diabetes and Exercise Metabolism, Arizona College of Osteopathic Medicine, Midwestern University, Glendale, AZ USA; 4 Department of Anatomy, Arizona College of Osteopathic Medicine, Midwestern University, Glendale, AZ 85308 USA

## Abstract

**Background:**

Obese, type two diabetics are at an increased risk for fracturing their limb bones in comparison to the general population. Phytoestrogens like as the soy isoflavone genistein have been shown to protect against bone loss. In this study, we tested the effects of genistein treatment on femurs of ob/ob mice, a model for obesity and type two diabetes mellitus.

**Methods:**

Twenty six-week-old female mice were divided into obese (ob/ob) control, obese genistein-treated, lean (ob/+) control, and lean genistein-treated groups (*n* = 5 each). Treatment with genistein consisted of 600 mg genistein/kg diet. Control mice were given standard rodent chow. At the end of a four-week treatment period, bone histomorphometric and three-point bending properties were compared among groups.

**Results:**

Obese mice had larger bone areas (B.Ar.; *P* < 0.05) and total areas (Tt.Ar.; *P* < 0.05), but similar bone volume (B.Ar./Tt.Ar.; *P* > 0.05) of the proximal femoral epiphysis in comparison to lean mice. Treatment with genistein decreased Tt.Ar. and femur length, and increased ultimate force required to fracture the femur and the maximum deformation to failure (*P* < 0.05).

**Conclusions:**

Genistein improves resistance to fracture from bending loads.

**Electronic supplementary material:**

The online version of this article (doi:10.1186/s12902-016-0144-4) contains supplementary material, which is available to authorized users.

## Background

Diabetes is a widely prevalent disease affecting approximately 8.5% of the population of the United States. It frequently presents with a variety of complications including hypertension, dyslipidemia, myocardial infarction, stroke, and kidney failure [1]. Obesity is a risk factor for Type 2 Diabetes Mellitus (T2DM), which is associated with an increased risk of limb bone fractures that take longer to heal and are more likely to result in infection and complications [2–4]. Recent evidence suggests T2DM reduces resistance to bending loads due to inefficient redistribution of bone mass [5].

Leptin is a hormone that is secreted by white adipose tissue to aid in the regulation of obesity by inducing weight loss and homeostasis of bone. Although obesity alone is not linked with decreases in bone mass [6], obese individuals with T2DM often exhibit leptin resistance that likely plays a role in increased incidence of fractures [7–10]. The ob/ob mouse is leptin deficient due to a spontaneous mutation of the ob gene. This model of obesity is a close counterpart to the human condition, resulting in hyperphagia, obesity, and a diabetes-like phenotype including insulin resistance, hyperglycemia, and decreased limb bone mass. As an animal model, the ob/ob mouse is commonly used to study the T2DM phenotype and leptin resistance characteristic of obesity and T2DM, particularly when associated with metabolic syndrome [11–15]. While metabolic syndrome alone is not associated with increased fracture risk, it is when found in conjunction with T2DM [13, 16, 17].

The objective of this study was to assess the effects of treatment with genistein on femoral bone structure and resistance to fracture in female ob/ob mice. Genistein is a phytoestrogen found in soybeans and soy-based foods. It is structurally similar to estrogen and can bind to estrogen receptors with great affinity [18, 19]. Phytoestrogens like genistein may prevent the reduction in bone loss in a manner similar to estrogen replacement therapy in postmenopausal women, but its full effects in the obese diabetic mouse model are unknown [19–24].

## Methods

### Experimental design

Twenty, female obese ob/ob mice and lean ob/+ mice (B6.V-Lep/J, Jackson Laboratory; Bar Harbor, ME, USA) aged six weeks were used in the experiment. Mice were kept in an animal facility with a 12 h light/dark cycle and temperature of 22 °C. Mice were given food and water ad libitum. These animal studies were approved by the Institutional Animal Care and Use Committee at Midwestern University and carefully followed the National Institutes of Health’s Guide for the Care and Use of Laboratory Animals.

At the commencement of the experiment, mice were divided into four groups of equal size (*n* = 5), (1) lean mice fed a standard diet, (2) lean mice fed a genistein diet, (3) obese mice fed a standard diet, and (4) obese mice fed a genistein diet. Genistein diet was formulated by Dyets Inc., (Bethlehem, PA, USA) and included 600 mg genistein/kg diet administered for the study period of 4 weeks. This diet is commonly used to study the effects of phytoestrogens on the T2DM condition in mouse models is comparable to human soy-based diets and has been shown to have significant physiological effects with four weeks of treatment [22, 25–28].

### Histomorphometry of the femur

After sacrifice, the hind limbs of each mouse were removed. Right and left femurs were harvested and dissected clean of soft tissue. One femur from each mouse was set aside for three-point bending testing. The other femur was used to conduct histomorphometric analysis of the proximal epiphysis and diaphysis. These femurs were dehydrated in 70 and 85% alcohol with two changes lasting 24 h in each concentration, cleared using Histoclear (National Diagnostics, Atlanta, Georgia, USA) in two 24 h washes, infiltrated with Osteo-Bed Resin A and catalyzed Osteo-Bed Resin A (100 mL Osteo-Bed Resin A, 1.40 g Benzoyl Peroxide) with two changes lasting 24 h, and then embedded in resin (100 mL Osteo-Bed Resin A, 3.50 g Benzoyl Peroxide; Polysciences Inc., Warrington, PA, USA). Six ml of embedding solution were added to vials containing the femurs, which were capped and placed in a bead bath at 33.5 °C for 48 h to polymerize. The position and orientation of the femurs in the vials was standardized to allow consistent orientation during sectioning.

After the resin hardened, a single section of the proximal femur was taken in the coronal plane and another was taken of the diaphysis in the transverse plane distal to the third trochanter using a low speed saw (Isomet; Buehler, Lake Bluff, IL, USA). Sections were cut at 200 μm in thickness, polished (MetaServ; Buehler, Lake Bluff, IL, USA) and stained with Alizarin Red (Sigma-Aldrich, Co., St Louis, MO, USA). Sections were then imaged at 40X magnification with an Eclipse 55i microscope (Nikon, Inc., Melville, NY, USA). ImageJ v1.6 (NIH) was used to measure total area (Tt.Ar.), bone area (B.Ar.), and bone volume (B.Ar./Tt.Ar.) for each proximal epiphysis based on Alizarin Red staining of bone tissue. The MomentMacroJ plugin (M Warfel and S Serafin) for ImageJ was used to calculate cortical area (Ct.Ar), maximum and minimum second moments of area (IMAX and IMIN) and the polar moment of area (J). These are measurements based on engineering beam theory that approximate resistance to compression, bending, and torsion from cross-sectional geometry.

### Three-point bending test of the femur

One femur from each specimen was subjected to a three-point bending test until failure to assess fracture resistance. Prior to loading, the length of each femur was measure to the nearest hundredth of a millimeter using digital calipers and the location of the midshaft was marked in pencil. Force was applied to the midshaft in the anterior-posterior direction at 0.5 N/s using a tip with a rounded edge (HP-5 with HSV Test Stand; Handpi Instruments Co., Ltd, China). Femurs were held on two supports that were positioned to contact the proximal and distal ends of the bone. The distance between supports was not held constant because femur length varied greatly in our sample and measurements of material properties of bone are proportional to the distance between the supports and the diameter of the bone in the breaking plane [29, 30]. Data on ultimate force, maximum displacement until failure, and time to failure were recorded. The location of the fracture expressed as a percentage of the total femur length along the long axis was also measured.

### Statistical analysis

Statistical analysis was completed using SPSS 19 software (IBM, USA). Two-way analysis of variance (ANOVA) tests were used to detect significant differences between treatment groups. Because bone fracture strength is proportional to bone length and diameter, the two-way ANOVA was repeated with femur length and diameter as covariates [24]. Statistical significance was set at *P* < 0.05 for all analyses. Tests of power, normality and homogeneity of variance show our analyses have adequate power to avoid type II errors and do not violate assumptions of the statistical analyses. The dataset is available in the “Additional files” section (Additional file 1: Genistein Dataset).

## Results

### Genistein treatment decreased body mass

Obese mice had greater body mass at the start of the experiment and at the time of sacrifice than lean mice (*P* < 0.05, Table 1). Mice fed 600 mg genistein/kg diet for four weeks had reduced body mass in comparison to control mice fed standard chow (*P* < 0.05). There was no significant genotype * treatment interaction (*P* > 0.05).

**Table 1:** Histomorphometry of the femur two-way analysis of variance

	Lean STD	Lean + GEN	Obese STD	Obese + GEN	Genotype effect (*P*)	Treatment effect (*P*)	Genotype * treatment interaction (*P*)
Starting body mass (g)	22.1 ± 0.92	21.7 ± 0.53	37.0 ± 1.86	36.54 ± 1.11	0.01	0.73	0.99
Final body mass (g)	24.8 ± 0.92	23.4 ± 0.46	50.7 ± 2.28	43.7 ± 1.29	0.01	0.01	0.06
PE Bone area (B.Ar.; mm^2^)	0.79 ± 0.15	0.50 ± 0.07	0.85 ± 0.06	0.84 ± 0.04	0.04	0.11	0.12
PE Total area (Tt.Ar.; mm^2^)	1.46 ± 0.26	0.84 ± 0.13	1.64 ± 0.17	1.44 ± 0.09	0.04	0.03	0.25
MS Cortical area (Ct.Ar.; mm^2^)	1.15 ± 0.06	1.20 ± 0.06	1.17 ± 0.03	1.08 ± 0.07	0.36	0.70	0.22
MS IMAX (mm^4^)	2.17 ± 0.20	2.11 ± 0.25	2.06 ± 0.12	1.98 ± 0.27	0.59	0.75	0.95
MS IMIN (mm^4^)	1.07 ± 0.12	1.29 ± 0.07	1.25 ± 0.06	1.09 ± 0.13	0.91	0.77	0. 09
MS J (mm^4^)	3.24 ± 0.31	3.40 ± 0.31	3.31 ± 0.17	3.07 ± 0.40	0.67	0.90	0.53

Data displayed as mean ± SE

*STD* fed standard chow; *GEN* fed 600 genistein/kg

*PE* proximal epiphysis of the femur; *MS* midshaft of the femur

*IMAX* maximum second moment of area; *IMIN* minimum second moment of area; *J* polar moment of area

### Genistein treatment decreased the total area of the proximal epiphysis of the femur but not the volume of bone

**Fig. 1:**
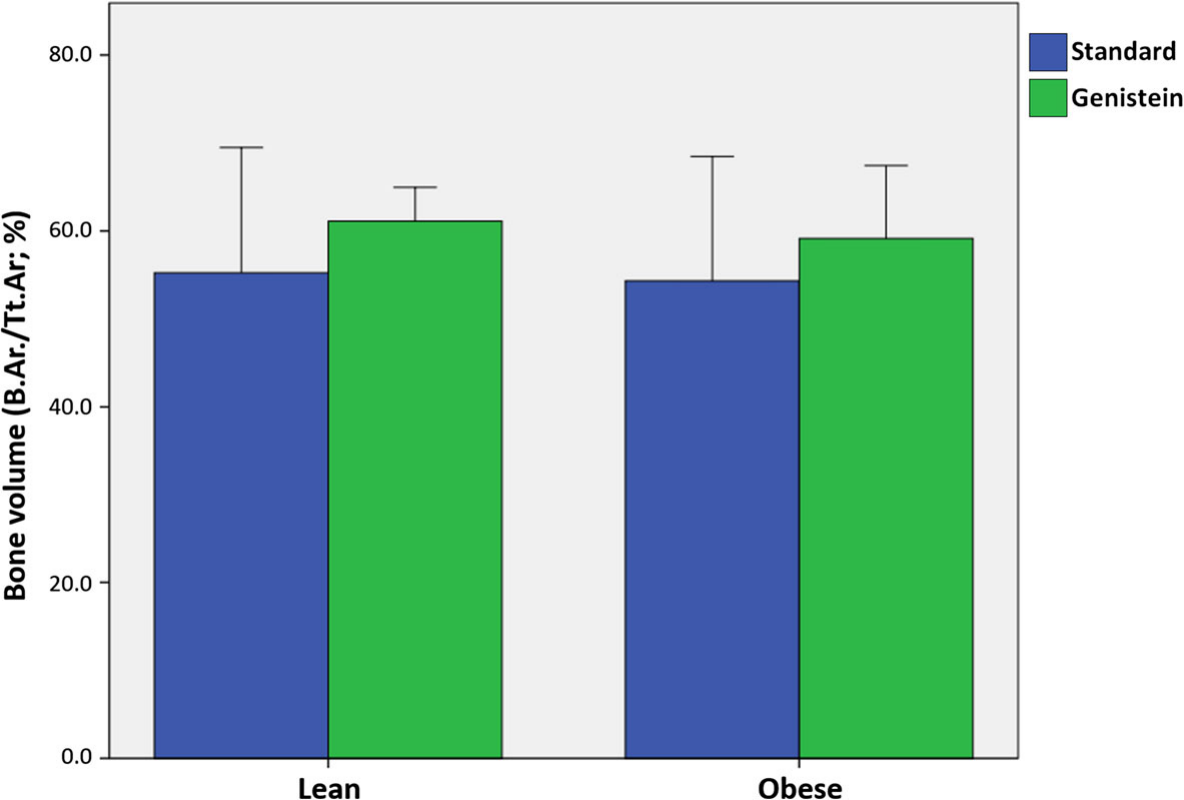
Comparison of ratios of bone area to total area (B.Ar./Tt.Ar.) of the proximal femoral epiphysis for lean and obese mice fed standard rodent chow or 600 mg genistein/kg diet. No genotype (ob/+ vs ob/ob) or treatment (standard vs genistein diet) effect was found with two-way analysis of variance (*P* > 0.05). Error bars are 2 ± SE

Obese mice had larger bone areas (B.Ar.) and total areas (Tt.Ar.) of the proximal femur than lean mice (*P* < 0.05, Table 1). However, bone volume (B.Ar./Tt.Ar.) was similar in lean and obese mice (*P* > 0.05, Fig. 1). Genistein treatment decreased the total area (*P* < 0.05), but had no effect on bone area or bone volume (*P* > 0.05). No interactions between genotype and treatment were found for these histomorphometric variables (*P* > 0.05).

### Genistein treatment had no effect on histomorphometric measures of bone strength

Comparisons of cortical area (Ct.Ar.), maximum and minimum moments of inertia (IMAX, IMIN), and polar moment of area (J) between lean and obese mice showed no differences (*P* > 0.05, Table 1). Comparisons of these variables between mice fed a standard diet and those treated with genistein also showed no differences (*P* > 0.05). No genotype * treatment interactions were found for any of the variables (*P* > 0.05).

### Genistein treatment decreased the length of the femur

**Fig. 2:**
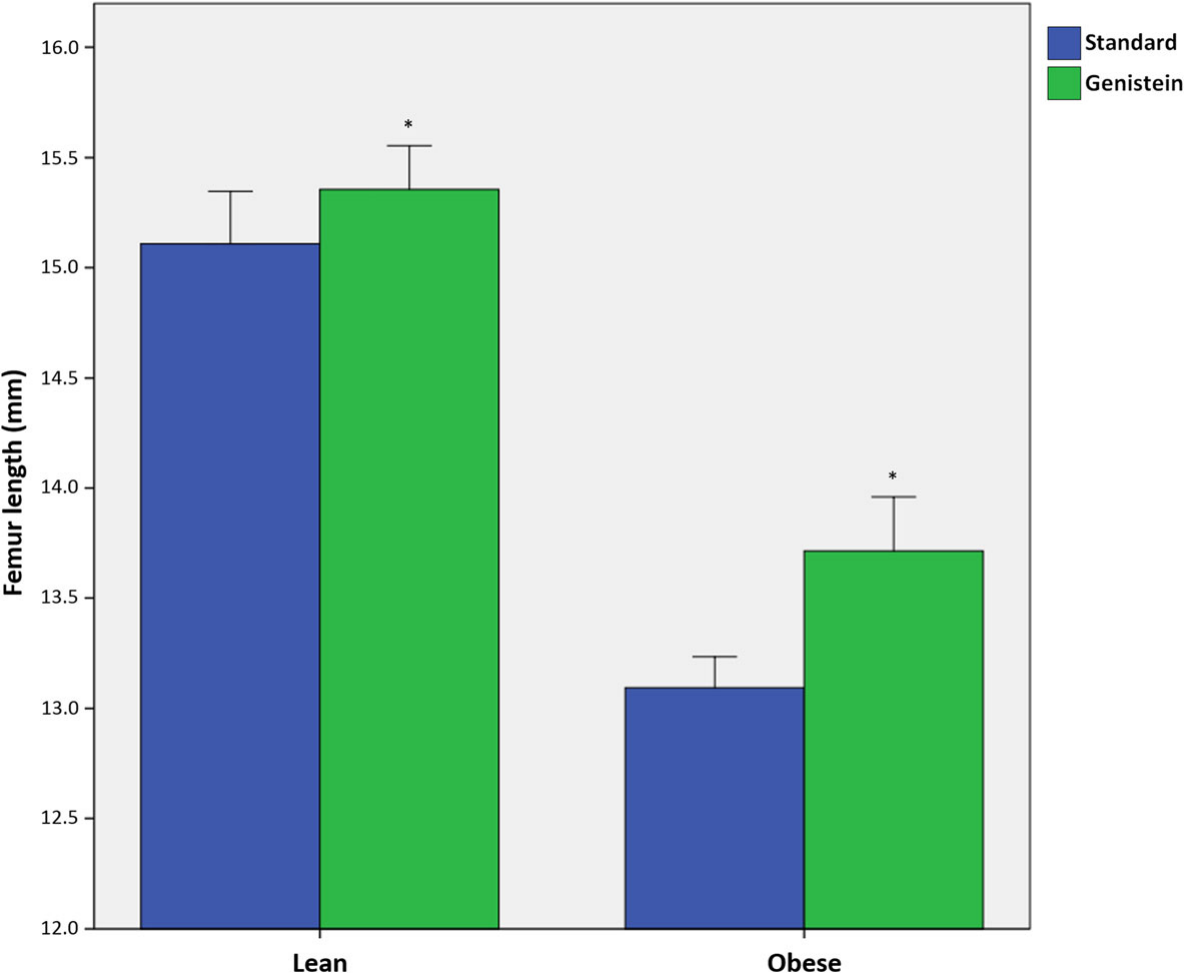
Comparison of femur length for lean and obese mice fed standard rodent chow or 600 mg genistein/kg diet. Mice treated with a genistein diet had significantly shorter femurs (*P* < 0.05). Femur length was also significantly reduced in obese mice in comparison to lean mice (*P* < 0.05). There was no genotype * treatment interaction (*P* > 0.05). Error bars are 2 ± SE

Femurs of lean mice were significantly longer than those of obese mice (*P* < 0.05, Fig. 2). Treatment with genistein significantly decreased femur length relative to mice fed standard chow (*P* < 0.05). For mice fed a standard diet, femurs of obese mice were 12.3% shorter than lean mice on average. Treatment with genistein increased this difference to 14.1%, although no interaction between genotype and treatment were found for femur length (*P* > 0.05).

### Genistein treatment increased the amount of force needed to fracture the femur

**Fig. 3:**
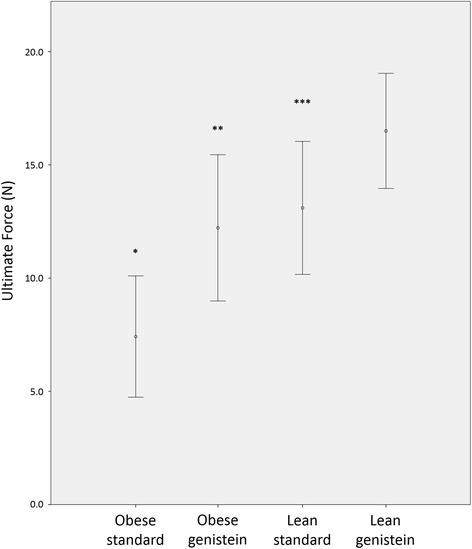
Comparison of ultimate load for lean and obese mice fed standard rodent chow or 600 mg genistein/kg diet. Treatment with genistein significantly increased the ultimate force to failure at the femoral midshaft (*P* < 0.01). There was also a significant genotype main effect. Lean mice had a significantly greater ultimate force in comparison to obese mice (*P* < 0.01). There was no interaction between treatment and genotype (*P* > 0.05). Error bars are 2 ± SE

**Fig. 4:**
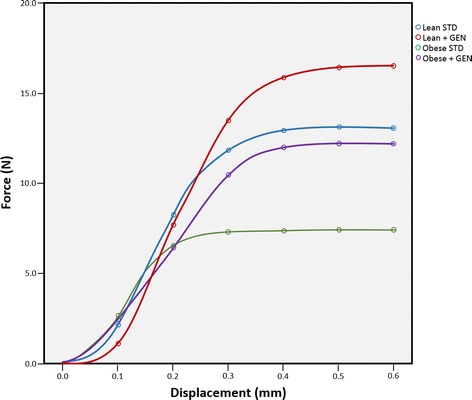
Load-displacement curves derived from three-point bending test results. The test was performed on femora of lean mice fed a standard diet (Lean STD), lean mice fed 600 mg genistein/kg diet (Lean + GEN), obese mice fed a standard diet (Obese STD), and obese mice fed 600 mg genistein/kg diet (Obese + GEN)

Results of the two-way ANOVA for the three-point bending test are shown in Table 2. Mice treated with genistein had femurs that were more resistant to fracture from bending loads at the midshaft. Ultimate force was significantly greater in genistein fed mice than mice fed standard chow (*P* < 0.05, Fig. 3). There was also a significant genotype effect. Femurs of lean mice had a greater ultimate force than obese mice (*P* < 0.05). Mice treated with genistein also exhibited greater maximum deformation to failure (*P* < 0.05). There were no genotype * treatment interactions for variables in this analysis (*P* > 0.05), indicating a genistein diet effects fracture resistance in both genotypes similarly. When the analysis was repeated with femur length and midshaft anterior-posterior diameter as covariates, the significant treatment main effect remained (*F* = 4.40; *P* < 0.05). However, there was no genotype effect nor genotype * treatment interaction (*F* = 0.45, *P* = 0.52; *F* = 0.33, *P* = 0.57, respectively). Load–displacement curves derived from three-point bending tests are displayed in Fig. 4.

**Table 2:** Three point bending of the femur two-way analysis of variance

	Lean STD	Lean + GEN	Obese STD	Obese + GEN	Genotype effect (*P*)	Treatment effect (*P*)	Genotype * treatment interaction (*P*)
Midshaft A-P diameter (mm)	2.14 ± 0.05	2.19 ± 0.07	2.09 ± 0.02	2.15 ± 0.05	0.42	0.32	0.86
Ultimate force (N)	13.1 ± 1.47	16.5 ± 1.27	7.4 ± 1.34	12.2 ± 1.61	0.02	0.01	0.63
Deformation to failure (mm)	0.36 ± 0.02	0.52 ± 0.05	0.32 ± 0.02	0.46 ± 0.10	1.00	0.01	0.50
Time to failure (s)	3.00 ± 0.55	3.60 ± 0.24	2.60 ± 0.24	3.40 ± 0.68	0.53	0.16	0.83
Fracture location (% femur length)	48.3 ± 12.6	49.9 ± 9.4	38.4 ± 8.57	45.3 ± 10.0	0.49	0.69	0.80

Data displayed as mean ± SE

*STD* fed standard chow; *GEN* fed 600 genistein/kg

## Discussion

Leptin deficient ob/ob mice are obese and demonstrate a clinically-relevant phenotype of T2DM [9]. Leptin is a hormone involved in metabolism regulation and plays an important part in controlling food intake as well as in bone development [7]. Consequently, limb bone length of ob/ob mice is reduced in contrast to lean mice [8, 23, 31]. Results of our experiment were consistent with these reports as we found ob/ob mice had significantly shorter femurs. While the precise mechanism that inhibits longitudinal limb bone growth is unknown, genistein and other phytoestrogens have been demonstrated to effect the thickness, calcification, and chondrocyte proliferation of limb bone growth plates, as shown in previous studies [22, 23, 32, 33]. The effects of genistein treatment on bone length may be due to its inhibitory effects on growth plate cartilage. Further investigation is needed to elucidate the mechanism.

Interestingly, we found genistein treatment had no effect on histomorphometric indicators of bone strength of the femur (e.g., bone volume, cortical area, IMAX and IMIN), yet genistein treatment did increase the ultimate force at the femur midshaft. Genistein has a high affinity for estrogen receptors and has been suggested to promote osteoblast activity through activation of ER, p38MAPK-Runx2, and NO/cGMP pathways and inhibit osteoclastic activities through inducing osteoprotegerin inhibition [34]. Other studies have shown genistein significantly increases bone calcium retention and serum levels of markers of bone formation in the estrogen-depleted state to improve fracture resistance [35–38]. This demonstrates genistein substantially influences bone metabolism. Bone mineral density and bone histomorphometry have been shown to be unreliable when predicting in vivo bone strains and fracture rates [39, 40]. However, bone quality is an important factor of bone health to consider. Hormone replacement therapy, which has shown to decrease fracture risk, has also been shown to increase bone quality [41, 42]. Bone quality is a composite of various geometric and compositional factors that contribute to fracture resistance [43–45]. Although we did not test bone composition and molecular structure, our results suggest genistein treatment improved bone quality in a manner that increased fracture resistance as indicated by three-point bending tests. In particular, the increased deformation to failure we found with genistein treatment suggests bone quality has been improved. Bones that are able to withstand greater deformation before failure are able to better dissipate energy to resist fracture [46, 47]. This property is largely attributable to the geometric arrangement and bonds between collagen molecules [47–49]. Genistein, like estrogen, may affect these properties to impart greater fracture resistance. Further study is required to determine the exact effects of genistein on bone geometry and composition to fully explore this hypothesis. Such studies should include micro-CT data, given phytoestrogen treatment has been shown to prevent loss of three-dimensional bone microarchitecture [50, 51]. Volumetric data may further explain the improved resistance to bending demonstrated in genistein-treated samples. Additionally, approaches that highlight the osteoclast-inhibiting effects of phytoestrogens, such as TRAP staining, should be used to assess how phytoestrogenic suppressive effects on bone resorption correlate with fracture resistance.

## Conclusions

Mice treated with 600 mg genistein/kg diet exhibit greater resistance to fracture during three-point bending tests in comparison to control mice fed standard rodent chow. These data provide support for the hypothesis that phytoestrogen intake improves limb bone resistance to fracture, not only in lean mice, but also in obese mice that display the T2DM phenotype. Future research needs to focus on markers of bone quality to determine how genistein effects bone ultrastructure and material properties.

## Additional files

Additional file 1: Genistein Dataset. Data used in the analyses in this study. (XLSX 12 kb)
Additional file 2: Supplementary tables. (DOCX 35 kb)

## Abbreviations

B.Ar.: Bone area
Ct.Ar.: Cortical area
IMAX: Maximum second moment of area
IMIN: Minimum second moment of area
J: Polar moment of area
T2DM: Type 2 diabetes mellitus
Tt.Ar.: Total area
