# Supplementary material for: Genistein treatment improves fracture resistance in obese diabetic mice
Source: BMC Endocr Disord. 2017 Jan 9;17:1. doi: 10.1186/s12902-016-0144-4 (PMC5299772; doi:10.1186/s12902-016-0144-4)
Supplement: Additional file 2: — Supplementary tables. (DOCX 35 kb) [file 12902_2016_144_MOESM2_ESM.docx]

**Supplemental Tables**

Table S1. Starting body mass ANOVA

| **Tests of Between-Subjects Effects** | | | | | |
| --- | --- | --- | --- | --- | --- |
| Dependent Variable: Starting body mass (g) | | | | | |
| Source | Type III Sum of Squares | df | Mean Square | F | Sig. |
| Corrected Model | 1108.014 | 3 | 369.338 | 50.988 | .000 |
| Intercept | 17185.039 | 1 | 17185.039 | 2372.446 | .000 |
| Genotype | 1107.072 | 1 | 1107.072 | 152.835 | .000 |
| Treat | .942 | 1 | .942 | .130 | .723 |
| Genotype * Treat | .001 | 1 | .001 | .000 | .992 |
| Error | 115.898 | 16 | 7.244 |  |  |
| Total | 18408.951 | 20 |  |  |  |
| Corrected Total | 1223.912 | 19 |  |  |  |
|  | | | | | |

Table S2. Ending body mass ANOVA

| **Tests of Between-Subjects Effects** | | | | | |
| --- | --- | --- | --- | --- | --- |
| Dependent Variable: Ending Body Mass (g) | | | | | |
| Source | Type III Sum of Squares | df | Mean Square | F | Sig. |
| Corrected Model | 2798.547 | 3 | 932.849 | 93.955 | .000 |
| Intercept | 25397.065 | 1 | 25397.065 | 2557.942 | .000 |
| Genotype | 2668.974 | 1 | 2668.974 | 268.814 | .000 |
| Treat | 88.789 | 1 | 88.789 | 8.943 | .009 |
| Genotype * Treat | 40.784 | 1 | 40.784 | 4.108 | .060 |
| Error | 158.859 | 16 | 9.929 |  |  |
| Total | 28354.471 | 20 |  |  |  |
| Corrected Total | 2957.406 | 19 |  |  |  |
|  | | | | | |

Table S3. Bone area ANOVA

| **Tests of Between-Subjects Effects** | | | | | |
| --- | --- | --- | --- | --- | --- |
| Dependent Variable: Bone Area (B.Ar.; mm2) | | | | | |
| Source | Type III Sum of Squares | df | Mean Square | F | Sig. |
| Corrected Model | .405 | 3 | .135 | 3.502 | .040 |
| Intercept | 11.120 | 1 | 11.120 | 288.768 | .000 |
| LEANOBESE | .193 | 1 | .193 | 5.014 | .040 |
| STDGEN | .110 | 1 | .110 | 2.856 | .110 |
| LEANOBESE * STDGEN | .102 | 1 | .102 | 2.637 | .124 |
| Error | .616 | 16 | .039 |  |  |
| Total | 12.141 | 20 |  |  |  |
| Corrected Total | 1.021 | 19 |  |  |  |
|  | | | | | |

Table S4. Total area ANOVA

| **Tests of Between-Subjects Effects** | | | | | |
| --- | --- | --- | --- | --- | --- |
| Dependent Variable: Total area (Tt.Ar., mm2) | | | | | |
| Source | Type III Sum of Squares | df | Mean Square | F | Sig. |
| Corrected Model | 1.834 | 3 | .611 | 3.980 | .027 |
| Intercept | 36.146 | 1 | 36.146 | 235.367 | .000 |
| LEANOBESE | .777 | 1 | .777 | 5.057 | .039 |
| STDGEN | .837 | 1 | .837 | 5.449 | .033 |
| LEANOBESE * STDGEN | .220 | 1 | .220 | 1.434 | .248 |
| Error | 2.457 | 16 | .154 |  |  |
| Total | 40.436 | 20 |  |  |  |
| Corrected Total | 4.291 | 19 |  |  |  |
|  | | | | | |

Table S5. Bone volume ANOVA

| **Tests of Between-Subjects Effects** | | | | | |
| --- | --- | --- | --- | --- | --- |
| Dependent Variable: Bone volume (B.Ar./Tt.Ar., %) | | | | | |
| Source | Type III Sum of Squares | df | Mean Square | F | Sig. |
| Corrected Model | 155.343 | 3 | 51.781 | .338 | .798 |
| Intercept | 66008.721 | 1 | 66008.721 | 431.320 | .000 |
| LEANOBESE | 10.540 | 1 | 10.540 | .069 | .796 |
| STDGEN | 143.439 | 1 | 143.439 | .937 | .347 |
| LEANOBESE * STDGEN | 1.365 | 1 | 1.365 | .009 | .926 |
| Error | 2448.621 | 16 | 153.039 |  |  |
| Total | 68612.685 | 20 |  |  |  |
| Corrected Total | 2603.964 | 19 |  |  |  |
|  | | | | | |

Table S6. Cortical area ANOVA

| **Tests of Between-Subjects Effects** | | | | | |
| --- | --- | --- | --- | --- | --- |
| Dependent Variable: Cortical area (Ct.Ar., mm2) | | | | | |
| Source | Type III Sum of Squares | df | Mean Square | F | Sig. |
| Corrected Model | .044 | 3 | .015 | .888 | .468 |
| Intercept | 26.568 | 1 | 26.568 | 1624.167 | .000 |
| LEANOBESE | .015 | 1 | .015 | .903 | .356 |
| STDGEN | .002 | 1 | .002 | .150 | .704 |
| LEANOBESE * STDGEN | .026 | 1 | .026 | 1.612 | .222 |
| Error | .262 | 16 | .016 |  |  |
| Total | 26.873 | 20 |  |  |  |
| Corrected Total | .305 | 19 |  |  |  |
|  | | | | | |

Table S7. IMAX ANOVA

| **Tests of Between-Subjects Effects** | | | | | |
| --- | --- | --- | --- | --- | --- |
| Dependent Variable: Maximum second moment of area (IMAX, mm4) | | | | | |
| Source | Type III Sum of Squares | df | Mean Square | F | Sig. |
| Corrected Model | .001 | 3 | .000 | .140 | .935 |
| Intercept | .866 | 1 | .866 | 358.752 | .000 |
| LEANOBESE | .001 | 1 | .001 | .311 | .585 |
| STDGEN | .000 | 1 | .000 | .104 | .751 |
| LEANOBESE * STDGEN | 9.522E-6 | 1 | 9.522E-6 | .004 | .951 |
| Error | .039 | 16 | .002 |  |  |
| Total | .906 | 20 |  |  |  |
| Corrected Total | .040 | 19 |  |  |  |
|  | | | | | |

Table S8. IMIN ANOVA

| **Tests of Between-Subjects Effects** | | | | | |
| --- | --- | --- | --- | --- | --- |
| Dependent Variable: Minimum second moment of area (IMIN, mm4) | | | | | |
| Source | Type III Sum of Squares | df | Mean Square | F | Sig. |
| Corrected Model | .002 | 3 | .001 | 1.153 | .358 |
| Intercept | .276 | 1 | .276 | 535.620 | .000 |
| LEANOBESE | 7.528E-6 | 1 | 7.528E-6 | .015 | .905 |
| STDGEN | 4.419E-5 | 1 | 4.419E-5 | .086 | .773 |
| LEANOBESE * STDGEN | .002 | 1 | .002 | 3.357 | .086 |
| Error | .008 | 16 | .001 |  |  |
| Total | .286 | 20 |  |  |  |
| Corrected Total | .010 | 19 |  |  |  |
|  | | | | | |

Table S9. J ANOVA

| **Tests of Between-Subjects Effects** | | | | | |
| --- | --- | --- | --- | --- | --- |
| Dependent Variable: Polar moment of area (J, mm4) | | | | | |
| Source | Type III Sum of Squares | df | Mean Square | F | Sig. |
| Corrected Model | .003 | 3 | .001 | .210 | .888 |
| Intercept | 2.121 | 1 | 2.121 | 446.437 | .000 |
| LEANOBESE | .001 | 1 | .001 | .191 | .668 |
| STDGEN | 8.487E-5 | 1 | 8.487E-5 | .018 | .895 |
| LEANOBESE * STDGEN | .002 | 1 | .002 | .421 | .526 |
| Error | .076 | 16 | .005 |  |  |
| Total | 2.200 | 20 |  |  |  |
| Corrected Total | .079 | 19 |  |  |  |
|  | | | | | |

Table S10. Femur length ANOVA

| **Tests of Between-Subjects Effects** | | | | | |
| --- | --- | --- | --- | --- | --- |
| Dependent Variable: Length of the femur (mm) | | | | | |
| Source | Type III Sum of Squares | df | Mean Square | F | Sig. |
| Corrected Model | 17.823 | 3 | 5.941 | 107.450 | .000 |
| Intercept | 4100.102 | 1 | 4100.102 | 74156.312 | .000 |
| Genotype | 16.708 | 1 | 16.708 | 302.187 | .000 |
| Treat | .942 | 1 | .942 | 17.033 | .001 |
| Genotype * Treat | .173 | 1 | .173 | 3.129 | .096 |
| Error | .885 | 16 | .055 |  |  |
| Total | 4118.810 | 20 |  |  |  |
| Corrected Total | 18.707 | 19 |  |  |  |
|  | | | | | |

Table S11. Femur diameter ANOVA

| **Tests of Between-Subjects Effects** | | | | | |
| --- | --- | --- | --- | --- | --- |
| Dependent Variable: Anterior-posterior diameter of the femur midshaft (mm) | | | | | |
| Source | Type III Sum of Squares | df | Mean Square | F | Sig. |
| Corrected Model | .024 | 3 | .008 | .590 | .630 |
| Intercept | 91.806 | 1 | 91.806 | 6861.444 | .000 |
| Genotype | .009 | 1 | .009 | .691 | .418 |
| Treat | .014 | 1 | .014 | 1.050 | .321 |
| Genotype * Treat | .000 | 1 | .000 | .030 | .864 |
| Error | .214 | 16 | .013 |  |  |
| Total | 92.044 | 20 |  |  |  |
| Corrected Total | .238 | 19 |  |  |  |
|  | | | | | |

Table S12. Ultimate force ANOVA

| **Tests of Between-Subjects Effects** | | | | | |
| --- | --- | --- | --- | --- | --- |
| Dependent Variable: Ultimate force to failure of the femur at midshaft (N) | | | | | |
| Source | Type III Sum of Squares | df | Mean Square | F | Sig. |
| Corrected Model | 210.502 | 3 | 70.167 | 6.871 | .003 |
| Intercept | 3030.722 | 1 | 3030.722 | 296.772 | .000 |
| Genotype | 124.002 | 1 | 124.002 | 12.142 | .003 |
| Treat | 84.050 | 1 | 84.050 | 8.230 | .011 |
| Genotype * Treat | 2.450 | 1 | 2.450 | .240 | .631 |
| Error | 163.397 | 16 | 10.212 |  |  |
| Total | 3404.621 | 20 |  |  |  |
| Corrected Total | 373.899 | 19 |  |  |  |
|  | | | | | |

Table S13. Deformation to failure ANOVA

| **Tests of Between-Subjects Effects** | | | | | |
| --- | --- | --- | --- | --- | --- |
| Dependent Variable: Ultimate displacement (mm) to failure | | | | | |
| Source | Type III Sum of Squares | df | Mean Square | F | Sig. |
| Corrected Model | .208 | 3 | .069 | 4.267 | .022 |
| Intercept | 3.872 | 1 | 3.872 | 238.277 | .000 |
| Genotype | .000 | 1 | .000 | .000 | 1.000 |
| Treat | .200 | 1 | .200 | 12.308 | .003 |
| Genotype * Treat | .008 | 1 | .008 | .492 | .493 |
| Error | .260 | 16 | .016 |  |  |
| Total | 4.340 | 20 |  |  |  |
| Corrected Total | .468 | 19 |  |  |  |
|  | | | | | |

Table S14. Time to failure ANOVA

| **Tests of Between-Subjects Effects** | | | | | |
| --- | --- | --- | --- | --- | --- |
| Dependent Variable: Time (s) to failure | | | | | |
| Source | Type III Sum of Squares | df | Mean Square | F | Sig. |
| Corrected Model | .030 | 3 | .010 | .894 | .466 |
| Intercept | 1.985 | 1 | 1.985 | 180.409 | .000 |
| Genotype | .004 | 1 | .004 | .409 | .531 |
| Treat | .024 | 1 | .024 | 2.227 | .155 |
| Genotype * Treat | .000 | 1 | .000 | .045 | .834 |
| Error | .176 | 16 | .011 |  |  |
| Total | 2.190 | 20 |  |  |  |
| Corrected Total | .205 | 19 |  |  |  |
|  | | | | | |

Table S15. Fracture location ANOVA

| **Tests of Between-Subjects Effects** | | | | | |
| --- | --- | --- | --- | --- | --- |
| Dependent Variable: Percent along the length of the femur where fracture occurred (%) | | | | | |
| Source | Type III Sum of Squares | df | Mean Square | F | Sig. |
| Corrected Model | 386.733 | 3 | 128.911 | .245 | .864 |
| Intercept | 41344.164 | 1 | 41344.164 | 78.554 | .000 |
| Genotype | 263.188 | 1 | 263.188 | .500 | .490 |
| Treat | 89.736 | 1 | 89.736 | .170 | .685 |
| Genotype * Treat | 33.809 | 1 | 33.809 | .064 | .803 |
| Error | 8421.052 | 16 | 526.316 |  |  |
| Total | 50151.949 | 20 |  |  |  |
| Corrected Total | 8807.785 | 19 |  |  |  |
|  | | | | | |
